# Assessment of single and double coronary bifurcation stenting techniques using multimodal imaging and 3D modeling in reanimated swine hearts using Visible Heart® methodologies

**DOI:** 10.1007/s10554-021-02240-0

**Published:** 2021-05-16

**Authors:** Thomas F. Valenzuela, Francesco Burzotta, Tinen L. Iles, Jens F. Lassen, Paul A. Iaizzo

**Affiliations:** 1 Department of Biomedical Engineering, University of Minnesota, Minneapolis, MN USA; 2 Department of Surgery, University of Minnesota, Minneapolis, MN USA; 3 Department of Bioinformatics and Computational Biology, University of Minnesota, Minneapolis, MN USA; 4 Institute for Engineering in Medicine, University of Minnesota, Minneapolis, MN USA; 5 Dipartimento di Scienze Cardiovascolari, Fondazione Policlinico Universitario A. Gemelli IRCCS, Università Cattolica del Sacro Cuore, L.go A. Gemelli 1, 00168 Rome, Italy; 6 Università Cattolica del Sacro Cuore, Milan, Italy; 7 Department of Cardiology, University of Copenhagen, Copenhagen, Denmark

**Keywords:** Coronary, Bifurcation, OCT, TAP-stenting, Culotte, DK-Crush, Personalized medicine

## Abstract

Stent implantation in bifurcated coronary lesions is technically challenging so that procedural refinements are continuously investigated. Novel procedure modeling and intracoronary imaging techniques may offer critical insights on stent deformations and stent-wall interactions during bifurcation stenting procedures. Thus, we assessed coronary bifurcation stenting techniques using multimodal imaging and 3D modeling in reanimated swine hearts. Harvested swine hearts were reanimated using Visible Heart® methodologies and (under standard fluoroscopic guidance) used to test 1-stent (provisional and inverted provisional) and 2-stent (culotte, TAP and DK-crush) techniques on bifurcations within various coronary vessels using commercially available devices. Intracoronary angioscopy and frequency-domain optical-coherence-tomography (OCT) were obtained during the procedures. 3D OCT reconstruction and micro-computed tomography 3D modeling (post heart fixations) were used to assess stent deformations and stent-wall interactions. We conducted multiple stenting procedures and collected unique endoscopic and OCT images (and subsequent computational models from micro-CT) to assess stent deformations and device/wall interactions during different steps of bifurcation stenting procedures. Endoscopy, micro-CT and virtual reality processing documented that different 1- and 2-stent techniques, practiced according to experts’ recommended steps, achieve optimal post-intervention stent conformation. As compared with intra-procedural endoscopy, software-generated 3D OCT images accurately depicted stent deformations during 1-stent techniques. On the opposite, during more complex 2-stent techniques, some defects were appreciated at 3D OCT reconstruction despite optimal 2D OCT images. This study provided unique insights regarding both stent deformations occurring in the course of bifurcation stenting and the efficacy of OCT to visualize them.

**Supplementary Information:**

The online version contains supplementary material available at 10.1007/s10554-021-02240-0.

## Introduction

Atherosclerosis can develop anywhere within human coronary arteries and branching points are commonly affected, likely due to shear stress issues [1]. Consequently, ~ 15–20% of percutaneous coronary interventions (PCI) are performed on bifurcation coronary lesions [2]. Relative to the implantation of drug-eluting metallic stents, 1- or 2-stent techniques are the standard approaches for PCIs within bifurcation lesions [3–5]. These PCIs are often technically complex and associated with higher occurrence of adverse events, both short- and long-term [6]. Refinements in bifurcation stenting techniques and PCI optimizations have the potential to positively impact bifurcation PCI outcomes [4]. Thus, improved understanding of stent deformations and stent-vessel interactions during bifurcation stenting procedures should be of pivotal relevance. During the clinical practice, intravascular imaging might offer the possibility to assess details of bifurcation stenting procedures [4] and optical coherence tomography is actually regarded as the gold standard [7]. In the setting of procedure simulations, microcomputed tomography imaging is becoming a popular method to analyze stents implanted both in-vivo and in-silico models to assess restenosis [8] and CFD modeling [9], respectively. However, these methodologies have yet to be applied for the analysis of technique specific procedural steps.

In the present study, we used cutting-edge multimodal imaging and 3D modeling technologies to assess procedural steps of different stenting techniques using a unique simulation environment recognized to be highly promising for bifurcation PCI understanding [3, 10].

## Materials and methods

The Visible Heart Laboratories routinely perform translational experimental research, reanimating large mammalian hearts using a supporting apparatus that can sustain organ function for 6–8 h [11]. The feasibility of coronary bifurcations and stenting procedures in the Visible Heart Laboratories has previously been reported [10].

Briefly, two healthy 80–90 kg swine were anesthetized and intubated, and isoflurane was continuously administered to maintain a 1-to-1.5 minimum alveolar concentration (MAC). A standard cardioplegia protocol was induced with a high potassium solution; after rapid arrest and cooling, each heart was then explanted and cannulated. Next, the heart was set up on an ex vivo cardiac perfusion apparatus (Visible Heart® methodologies), where it was warmed by perfusion with a Krebs Henseleit buffer at 36.0 + 0.5 °C and reanimated with a 30 J defibrillatory shock (LifePack, Physio-Control, Redmond, WA, USA). Once hearts were reanimated and elicited native sinus rhythm, two board-certified interventional cardiologists performed various PCI techniques with multimodal visualizations. Commercially available guiding catheters, guidewires (Cougar XT, Medtronic, Santa Rosa, CA, USA), balloons (Euphora, Medtronic, Santa Rosa, CA, USA) and drug-eluting stents (Resolute Onyx, Medtronic, Santa Rosa, CA, USA) were utilized, under the guidance of standard fluoroscopy (OEC Elite Fluoroscopy, GE, Boston, MA, USA) and direct visualization.

The following techniques were performed based on best practice recommendations of the European Bifurcation Club (EBC) [5]:

- One-stent (provisional and “inverted” provisional), with a focus on specific steps including proximal optimization technique (POT) and kissing balloon inflation;
- Double-stenting with culotte;
- Double-stenting with T and small protrusion (TAP);
- Double-stenting with double-kissing (DK) crush.

Intracoronary angioscopy, using a 2.4 mm videoscopic camera (Olympus, Japan), was utilized during the procedures, keeping the probe immediately proximal (~ 1 to 1.5 cm) to the bifurcation. Overhead video cameras continuously captured relative heart function as well as the operator’s hands, to monitor how various devices, catheters, and wires were manipulated. Heart hemodynamics, such as left ventricular and aortic pressures, were also monitored throughout the study (EMKA Technologies, Paris, France). These modalities, in addition to optical coherence tomography (OCT) and fluoroscopy, were continuously recorded with a customized 6-channel digital recording system (Z Systems, Inc., St. Louis Park, MN, USA).

**Fig. 1:**
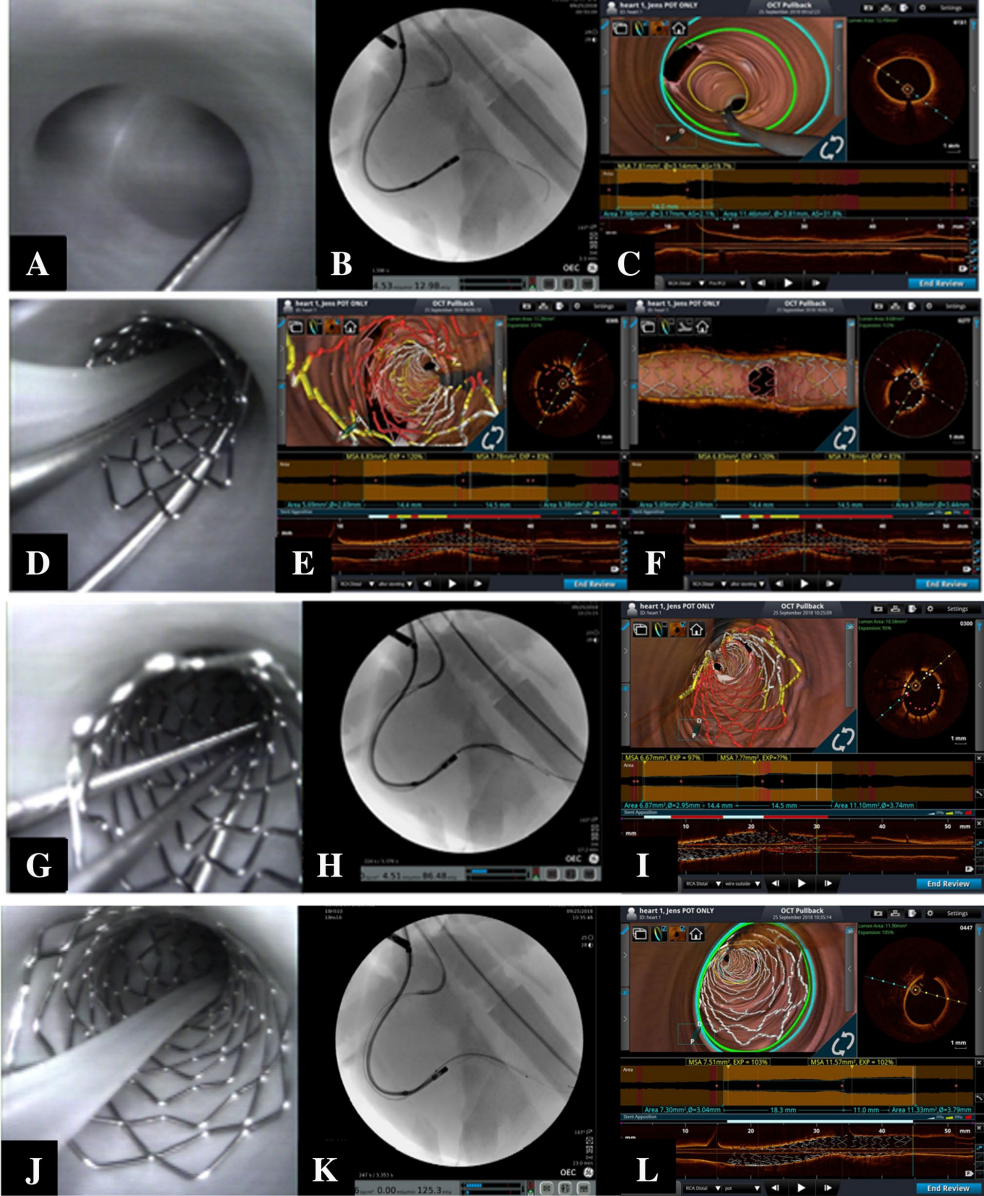
**a**–**c** Angioscopic visualization and 3D optical coherence tomography (OCT) reconstruction of a representative coronary bifurcation. **a** Angiographic image shows guidewire placement in descending posterior and angioscopy device in distal right coronary. **b** Distal right coronary bifurcation with guidewire in descending posterior artery. **c** Distal right coronary bifurcation obtained with 3D OCT reconstruction. **d**–**i** Multimodal assessment of results achieved after crossover stenting (stent sized according to distal main vessel) in provisional. Perfect 3D OCT reconstructions show stent malapposition in proximal main vessel, side branch “jailing” due to floating stent struts, and side branch ostium shape preservation. **d** Direct visualization of proximal malapposition of stent with OCT catheter following the main vessel guidewire. **e** Abluminal guide wire crossing is clearly identified in the 2-D OCT image. The 3-D OCT image is also able to provide useful information. **f** 3D OCT reconstruction of malapposed stent at the bifurcation as seen from the longitudinal axis. There is failure of incorrect guidewire tracking identification by 3D OCT reconstruction when side branch rewiring is attempted on top of grossly malapposed stent struts. **g** Direct visualization captured the guidewire tracking through stent struts. **h** The most common imaging modality used during percutaneous coronary intervention, fluoroscopy, is unable to detect this improper wiring. **i** Visualizing the guidewire rewiring through the proximal stent struts was difficult to interpret via the 3-D OCT image. **j** Angiographic image showing proper proximal stent apposition immediately following POT. **k** Change in apposition was not easily detected through fluoroscopy when compared to other visualization methods being used. **l** OCT reconstruction post POT shows proper apposition was achieved throughout the stent

Frequency-domain OCT was performed during the procedures, after each step, using Dragonfly OPTIS probes (Abbott Vascular, Abbott Park, IL, USA). All OCT pullbacks were taken at 54 mm scanning lengths with 5 mm penetration distance. Long-axis and 3D OCT reconstructions were completed using the metallic stent optimization tools of the OPTIS OCT Ilumien (Abbott Vascular). To highlight stent-vessel interactions, the apposition analysis tools were set according to the distance between the stent struts and vessel walls as follows: *white* when the estimated distance was < 200 µm, *yellow* when between 200 and 300 µm, and *red* when > 300 µm. As an example, Fig. 1a–c shows the angiographic screen, angioscopic image, and 3D OCT reconstruction of a distal right coronary artery bifurcation after main vessel (MV) wiring.

After the stenting procedures, hearts were perfusion fixed using a formalin fixation chamber [12] that preserved them in an end-diastolic state, keeping the coronaries well dilated. After fixation, hearts were scanned with an X5000 micro-CT scanner (North Star Imaging, Minneapolis, MN, USA) that allowed for computational stent reconstructions with approximately 20-micron resolution. From these scans, resulting stent(s) and anatomies were 3D reconstructed, segmented, and rendered using medical imaging software (Mimics, Materialise, Leuven, Belgium).

## Results

### One-stent technique: proximal optimization technique

According to EBC recommendations [3–5], the minimal treatment for bifurcated coronary lesions is stent implantation in the MV (also called “crossover” stenting) with the stent sized 1:1 according to the distal MV, followed by POT with the balloon sized 1:1 according to the proximal MV.

After crossover stenting, major malapposition in the proximal MV was systematically visualized by angioscopy, and well appreciated (and graded) by 3D OCT (Fig. 1d–f). Note that in this procedure, the side branch (SB) ostium shape was not affected, and 3D OCT documented floating stent struts “jailing” the SB take-off. For instance, when attempting to rewire the SB at this stage, the guidewire was easily advanced incorrectly across some malapposed stent struts. Such phenomena were not recognized by fluoroscopy, yet were clearly identified by the videoscopic imaging (Fig. 1g–i). Importantly, 3D OCT reconstructions were not able to correctly capture this event.

After POTs, completely changed stent conformations were consistently achieved. As shown in Supplementary Fig. 1, proper appositions were obtained throughout the stent, and both longitudinal and 3D OCT reconstructions demonstrated this new, more favorable stent conformation. Furthermore, significant enlargement of the SB cells (Supplementary Fig. 1C compared to Fig. 1g–i) was induced by the POT-associated stent expansion. Finally, post-procedure micro-CT scans confirmed these features that were also virtually inspected with virtual reality (Supplementary Fig. 1 E).

### One-stent technique: kissing balloon inflation

**Fig. 2:**
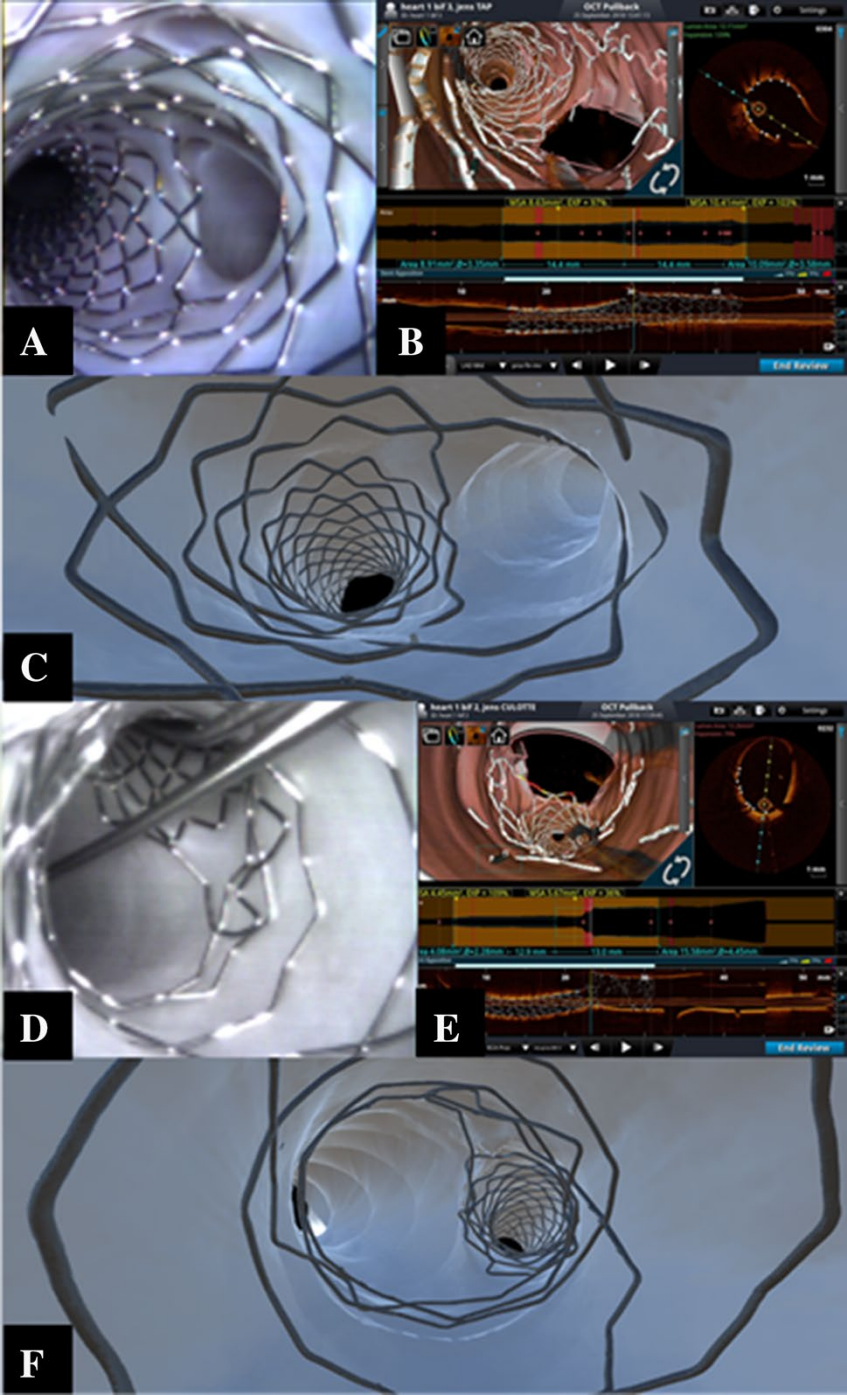
Multimodal assessment of results achieved after kissing and re-proximal optimization technique (POT) in provisional. Results of provisional bifurcation technique after a final POT as viewed by angioscopy (**a**), 3D optical coherence tomography reconstruction (**b**), and micro-CT models (**c**). Multimodal assessment of inverted provisional results: perfect inverse provisional visualized by direct angioscopy (**d**), 3D optical coherence tomography reconstruction (**e**), and micro-CT models (**f**)

During one-stent strategies, after stenting the MV, it may become clinically necessary to intervene on the SB. To do this, physicians typically perform SB rewiring, followed by kissing balloon inflation [3–5]. Since the SB rewiring site is known to influence final stent deformations [13], we performed side-cell crossing to the SB using a pullback technique [14] in order to facilitate “distal” stent frame rewiring. In our study, kissing balloon inflations were conducted with short non-compliant balloons sized 1:1 according to the distal MV and SB. Figure 2a–c shows multimodal assessments of the final results achieved in the left anterior descending artery/first diagonal bifurcation using the sequence of crossover stenting, POT, distal rewiring, kissing balloon inflation, and repeat POT (performed with a balloon sized 1:1 according to the proximal MV). All images collected by angioscopy, OCT, and micro-CT documented favorable stent conformation, with complete removal of stent struts from the SB ostium and absence of “oval deformation” in the proximal MV (Fig. 2a–c). Furthermore, optimized interactions between the stent and vessel wall were detected by apposition analyses, showing absence of malapposed areas (i.e., absence of yellow or red stent struts in Fig. 2a–c). It is important to note that the longitudinal OCT stent reconstructions were considered optimal, while some unrecognized struts that created (minor) imperfections, detected with micro-CT studies, in the 3D OCT were seldom noticed (see Fig. 2 around SB ostium).

In some specific anatomic conditions, the selection of an “inverted” provisional strategy might considered [3–5]. This technique is executed by implanting the stent from the SB into the proximal MV, then following the same steps of provisional (POT, distal rewiring, kissing, and re-POT). Potential drawbacks are: (1) the same stent might cover vessel segments with major size mismatches, and (2) side cells are required to be extensively dilated to achieve optimal stent removal from the distal MV ostia. The 3D OCT and micro-CT results of an inverted provisional (performed in right coronary artery bifurcation of a swine’s heart) is shown in Fig. 2d–f, showing wide opening of the stent struts toward the distal MV.

### Two-stent techniques: TAP stenting

**Fig. 3:**
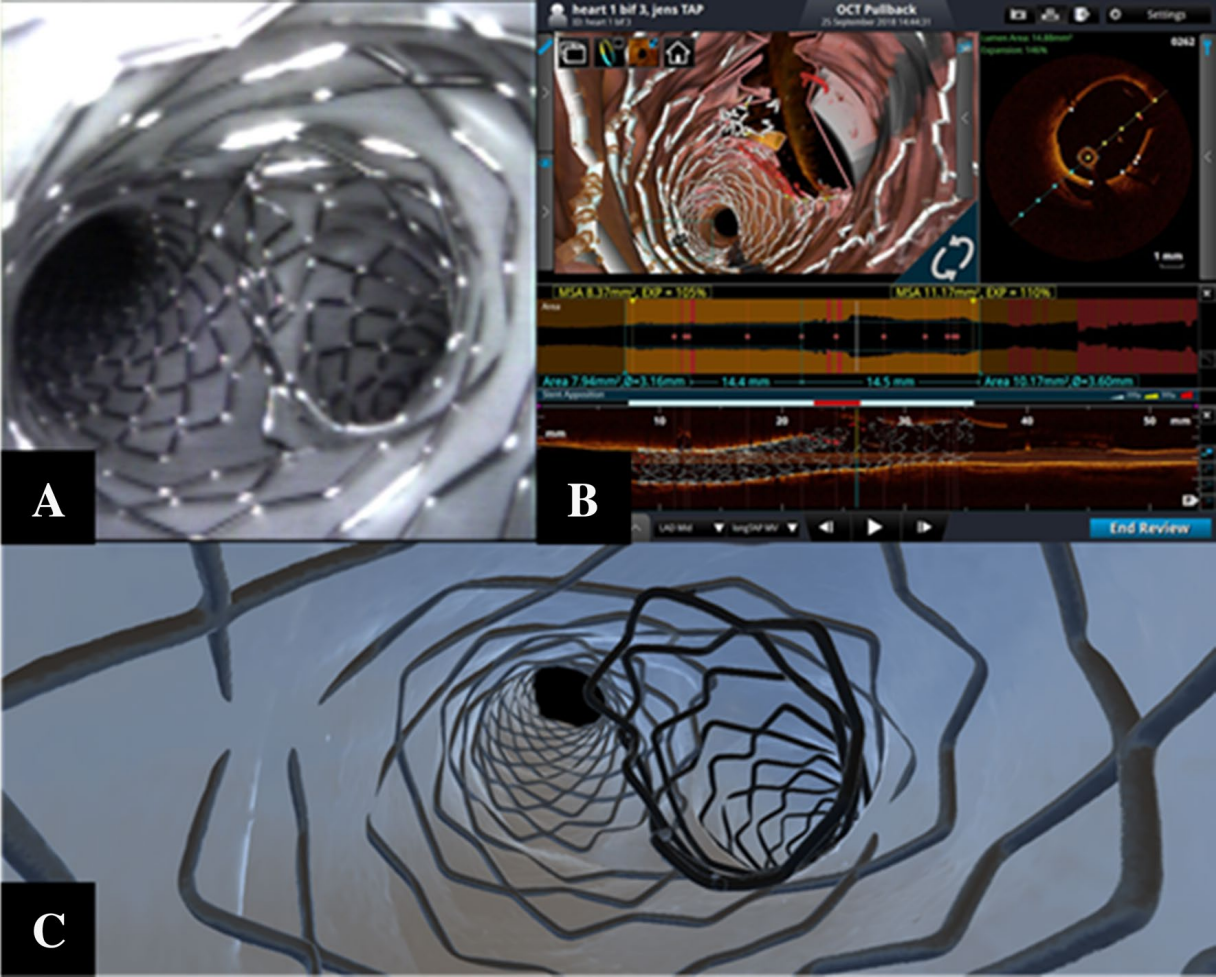
Multimodal assessment of T and protrusion (TAP) stenting results using angioscopy (**a**), 3D optical coherence tomography, or OCT (**b**), and micro-CT (**c**). With 3D OCT, the small neocarina was perfectly captured and represented the only red colored (malapposed strut coding) segment in the two implanted stents

A possible way to efficiently implant a SB stent in the course of provisional approach, is TAP-stenting technique [15]. In reanimated hearts, we performed the TAP technique following all steps previously reported for provisional (crossover stenting, POT, kissing, repeat POT). Then, a new balloon (sized 1:1 according to the distal MV) was advanced down the MV and a stent (sized 1:1 according to SB diameter) was advanced into the SB. After proper positioning to avoid long protrusion and ensure ostial coverage, we deployed the SB stent. Thereafter, the delivery balloon was pulled back and reinflated at a higher pressure to ensure proper stent expansion in the SB ostium. Once the SB stent was fully deployed, the stent delivery balloon and the balloon that was advanced previously in the main branch were used to perform a final kissing balloon technique, taking care to simultaneously deflate the balloons in order to maintain a central position for the new metallic carina. We then applied a final POT, making sure to avoid contact between the metallic neocarina and the balloon. Figure 3 shows the TAP results achieved, as assessed by angioscopy, 3D OCT, and micro-CT. Using 3D OCT, the small neocarina was perfectly captured, and represented the only red colored (malapposed strut coding) segment within the two implanted stents.

### Two-stent techniques: culotte technique

**Fig. 4:**
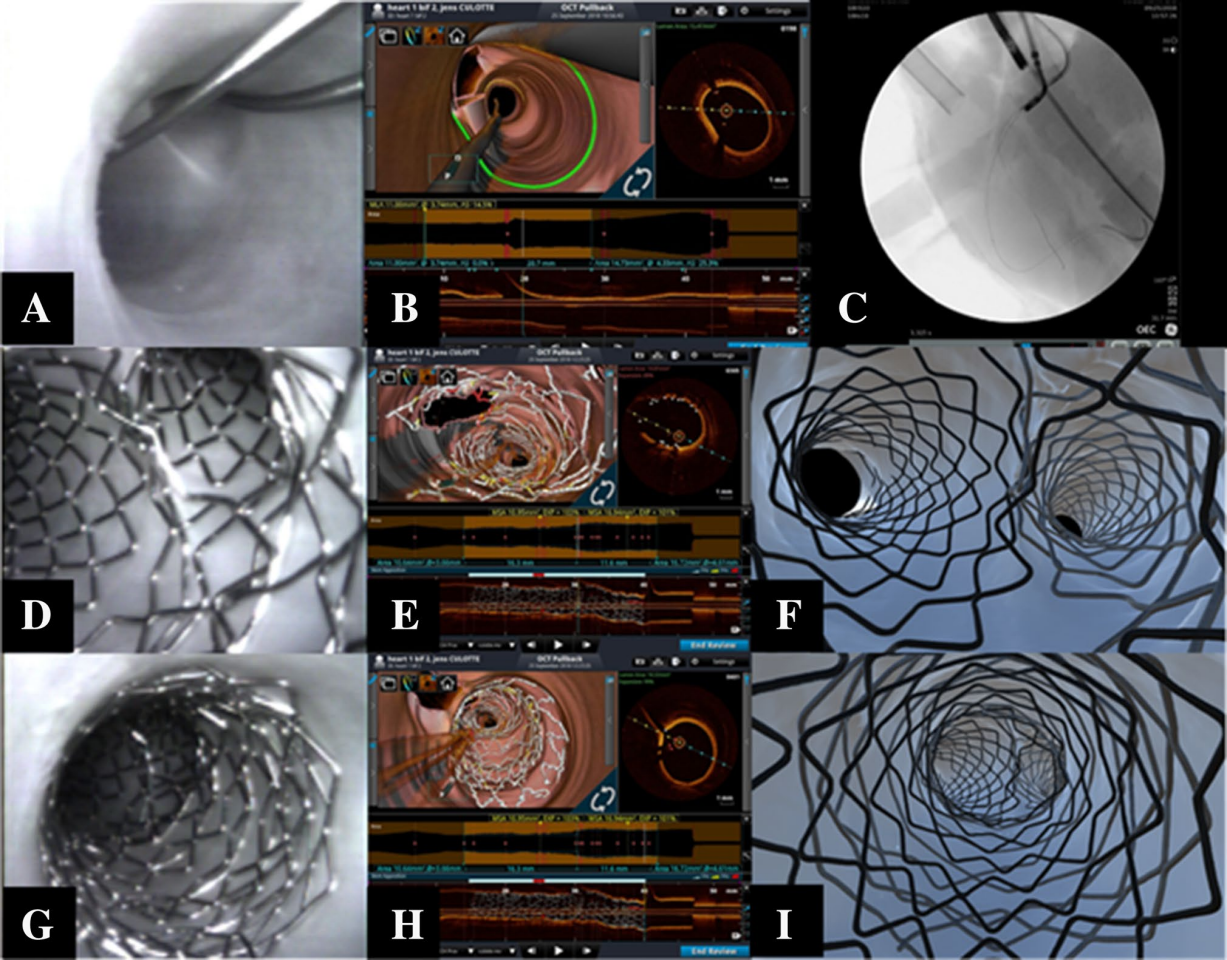
**a**–**c** Angioscopic visualization and 3D optical coherence tomography (OCT) reconstruction of a coronary bifurcation with large side branches. Fluoroscopic, direct, and 3D OCT reconstruction of a bifurcation in which anatomy is best suited for culotte. **a** Fluoroscopic view capturing guidewires, angioscope, and launcher. **b**, **c** Direct and OCT visualization of wired bifurcation before percutaneous coronary intervention. **d**–**i** Multimodal assessment of results achieved after culotte technique. Angioscopic (**d**), 3D OCT (**e**), and micro-CT images (**f**) highlighting the bifurcation and carina achieved from culotte procedure. Proximal portion of the culotte viewed by angioscopy (**g**), 3D OCT (**h**), and micro-CT (**i**). One can identify where the 3D OCT reconstruction is unable to accurately represent the proximal double stent layer

A culotte procedure may begin by deploying a stent in the MV (according to crossover stenting provisional approach) or covering the SB and proximal MV (according to inverted provisional approach) [3–5]. Regardless of beginning strategy, both stents must be appropriately sized to fit each of the distal branches as well as the proximal MV. Accordingly, a favorable anatomy for this technique is a bifurcation with two large branches. This anatomic requirement was evident in the coronary bifurcation shown in Fig. 4a–c, where a culotte was performed according to the “inverted” provisional strategy [15]. Thus, the first stent (sized 1:1 according to SB diameter) was implanted from the MV into the SB, and the first POT (balloon sized 1.1 according to proximal MV) was applied. Then, we performed distal MV rewiring (close to carina), followed by the first kissing balloon inflation. Next, a stent (sized 1.1 according to distal MV) was implanted into the MV across the SB take-off and a second POT was performed. We then conducted SB rewiring (to achieve “distal” crossing close to carina), followed by a second kissing balloon step and a third POT. The multimodal assessments of this inverted culotte are shown in Fig. 4d–i, presenting an optimal result via angioscopy and micro-CT. The 3D OCT reconstruction also captured the optimization of stent struts in the distal MV and SB, while proximal MV struts were not consistantly tracked.

### Two-stent technique: double-kissing crush

**Fig. 5:**
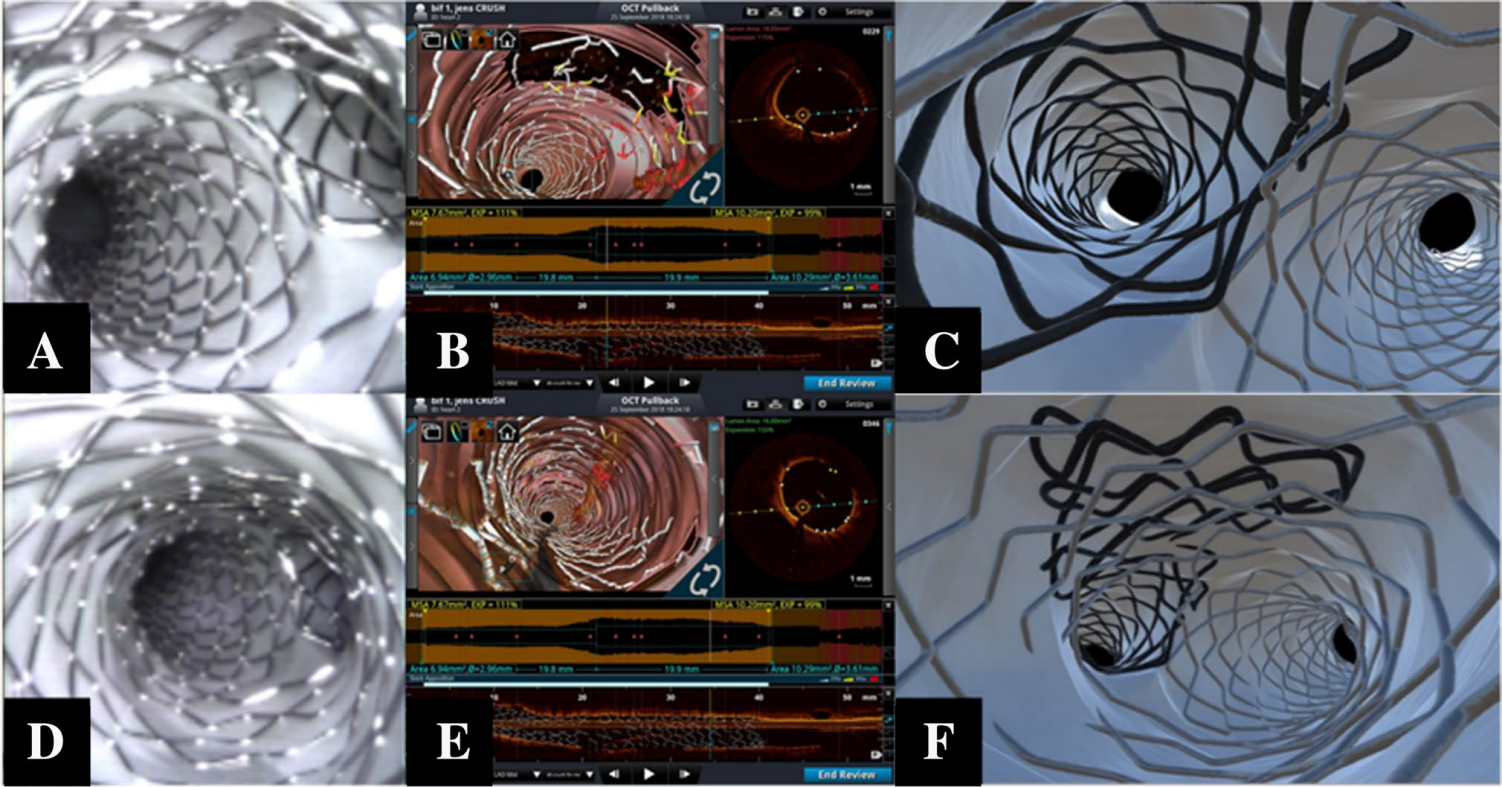
Multimodal assessment of results achieved after Double Kissing (DK)-crush technique**.** Angioscopic (**a**), 3D optical coherence tomography (OCT) reconstruction (**b**), and micro-CT (**c**) visualization of bifurcation and carina. OCT is able to properly depict the stent until multiple stent layers occur. Angioscopic (**d**), 3D OCT reconstruction (**e**), and micro-CT (**f**) visualization of the main vessel. Note that OCT is not able to register the stent where the crush occurred

The DK-crush represents another valuable two-stent technique aimed at first securing the SB. We performed this technique in reanimated swine hearts at a bifurcation in the mid left anterior descending artery, following the steps described by Chen et al. [16]. The SB stent was sized relative to the distal portion of the SB and was implanted protruding 2–3 mm into the MV. We also used a balloon sized according to the proximal MV to crush the stent after it was deployed. Figure 5 shows the results achieved after the initial balloon-crush. Indeed, the possibility of incomplete stent crush was clearly noticed (two layers of the SB stent apposed against the MV lumen), and could be appropriately corrected by further ballooning using a larger balloon. For instance, 3D OCT allowed to clearly recognize the degree of stent crushing in the proximal MV (Fig. 5). Of note, the occurrence of incomplete crush has been recently recognized as a potential pitfall and the possibility to use a POT balloon suggested [5]. Once an optimal crush was achieved, we completed non-distal SB rewiring followed by kissing. The remaining steps (MV stenting, POT, SB rewiring, and final kissing inflation) were then performed [5] to complete the procedure and achieving optimal endoscopic result. Importantly, some of the multiple, distorted layers of crushed stent were not entirely correctly captured by the automatic 3D OCT reconstruction.

Supplementary Fig. 2 illustrates the multimodal assessment of results achieved by the DK-crush technique. Angioscopy and micro-CT were both used to document an optimal result, whereas 3D OCT reconstruction performed by MV pullback revealed good results in the MV but not in the SB, where some “false” floating struts in the ostium appear to have been generated.

## Discussion

Refinements of bifurcation stenting techniques and their evaluation during clinical practice using imaging are hot topics of interventional cardiology. In the present study, we assessed various popular bifurcation stenting techniques within a unique ex vivo porcine model, using Visible Heart® methodologies, multimodal imaging, and advanced 3D reconstruction technologies. The main findings of our study include the following:

1. Angioscopy and micro-CT documented that different stenting techniques (applied according to recommended technical steps) are able to warrant favorable stent deformation in coronary bifurcations.
2. Cutting-edge 3D OCT modeling provides high-fidelity stent strut reconstructions in the simpler techniques allowing for immediate appraisal of important stent deformations (main vessel malapposition, side-cell size and opening etc.)
3. During more complex 2-stent techniques where stent deformations are more prominent, 3D OCT might exhibit inaccurate 3D renderings.

These findings support both the adoption of specific technical steps during stenting and the use of OCT imaging during bifurcation PCI. In the specific setting of complex 2-stent techniques, due to the occurrence of major stent distortions, 3D OCT reconstructions might exhibit deficiencies so that 2D OCT images have to be carefully assessed. Overall, the presented images might be used for educational initiatives and to prompt novel improvements in both bifurcation stenting techniques and in vivo imaging modalities.

### Insights regarding proximal optimization technique

In general, when performing any type of bifurcation procedure, it is considered critical to perform a POT immediately after the initial stent deployment, regardless if it is implanted in the main or side vessel [3–5]. Our multimodal imaging assessments documented that appropriately sized balloons for POT resulted in more favorable outcomes, and 3D OCT software was able to identify and quantify (through color coding) the achieved stent expansion and apposition. The wide malapposition that was present before POT may induce issues when attempting SB rewiring (Fig. 1g–i), if and when the guidewire passes through the first few malapposed stent struts. If unrecognized, such abluminal wire track may hinder balloon advancement and/or cause significant stent deformations. Intravascular imaging is an important tool to recognize such pitfalls, yet 3D OCT software reconstructions, in some cases, may fail to automatically detect the different metallic structures during various critical steps. Accordingly, when intravascular imaging is selected, physicians should pay meticulous attention to both cross-section reconstructions, and 3D software reconstructions should be performed with caution.

Another important finding in our study came from 3D assessment of the appearance of the stent’s side cells at the level of the SB ostium. We observed the same effects that Mortier et al. [17] reported in a bench simulation study: POT induces a favorable expansion of the distance between stent struts, and this reduced floating stent struts in the SB ostia and facilitated eventual advancement of guidewires and balloons in the SB.

### Insights on 1-stent techniques

Single-stent techniques continue to be regarded as the gold standard in most bifurcation cases and provisional or (less commonly) inverted provisional represent the main operative options [3–5]. Both techniques may be accomplished by selecting stents sized 1:1 according to the distal vessel to be stented, and engaging the main vessel for the standard provisional approach and the side vessel for the inverted provisional. Using multimodal imaging (and employing the latest generation drug-eluting stent), we clearly showed the MV stent being fully expanded, and optimal apposition after POT. Additionally, we observed side-cell expansion induced by kissing balloon, to achieve optimal results for both main and SB vessels. The primary difference between the traditional and inverted provisional techniques was represented by the metallic coverage of the main vessel—smaller in the inverted provisional method where a smaller stent size was implanted in the MV (Figs. 2, 3 and 4).

### Insights on 2-stent bifurcation techniques

In the present study, we utilized multimodal imaging to visualize three popular double stenting techniques: TAP, culotte, and DK-crush. In general, the TAP technique represents the simplest two-stent technique and the only method with anticipated certainty of final kissing performance. Yet, a well-recognized drawback of TAP is the potential creation of a metallic neocarina of variable lengths. Our multimodal assessments of TAP in the reanimated swine heart confirmed that 3D OCT software reconstructions allowed to recognize and visualize the metallic neocarina struts (Fig. 3).

The culotte procedure is a 2-stent technique that requires both stents to fully cover the lumen proximal to the bifurcation. As most recently recommended by EBC experts, as many as three POT and two kissing balloon techniques may be applied to facilitate a successful procedure [5]. Relative to this defined sequence, we obtained optimal results in reanimated swine hearts, as assessed by intra-procedure angioscopy and subsequent micro-CT analyses. Of note, the complex metallic coverage of the vessels by overlapping stent layers was not precisely captured by 3D OCT reconstructions. Furthermore, 3D OCT images from MV and SB deliveries and reconstructions elicited some differences in the proximal MV assessments (Fig. 4). More specifically, scans obtained from the SB (in this case, the branch where the stent was deployed) captured more stent struts proximal to the carina when compared to MV deployed scans.

The last bifurcation technique investigated was the DK-crush, a modification of the crush technique aimed at facilitating the final kissing inflation [16]. Recently, this technique is gaining acceptance due to promising clinical efficacy for complex distal left main lesions [18]. Our multimodal assessments showed that the early steps of proximal stent strut crushing by a MV balloon might be partial, and this may cause floating struts in the proximal MV. Importantly, this potential pitfall was easily detected by 3D OCT reconstructions and might explain the difficulties experienced clinically during the first SB rewiring and dilation. Accordingly, careful attention to routinely check the effectiveness of stent crushing and selecting appropriately sized balloons/pressures is advisable. The final results of the DK-crush achieved in our study were optimal, as assessed by live angioscopy and subsequent micro-CT reconstructions. Yet, the final 3D OCT reconstructions for this procedure looked fragmented, especially at the SB ostium.

### Computational modeling and educational uses of virtual reality

The detailed ~ 20 micron computational models generated from micro-CT reconstructions of perfusion-fixed specimens were also used for generating virtual reality scenes. For several years, the Visible Heart Laboratories have implemented virtual reality for medical education [19], and more recently ventured into the bifurcation stenting space. In one virtual scene, the user can “fly around” the coronary anatomies and deployed stent(s). Interventionalists, fellows, medical students, engineers and others have reported that these scenes allow one to acquire a deeper appreciation for the steps necessary to obtain successful stenting results. Flythrough animations of the micro-CT models used in this work (provisional, inverted provisional, TAP, culotte, and DK-crush) can be found in the “Atlas of Human Cardiac Anatomy,” a free-access educational website (www.vhlab.umn.edu/atlas/device-tutorial/stents/index) [20]. These virtual scenes are freely shared as educational tools for interventional cardiologists, all types of students, and device designers.

## Limitations

Although the present study allowed collecting original multimodal images with promising educational potential, a series of limitations have to be highlighted. We performed stenting procedures in a specific experimental setting (healthy swine coronary bifurcations [21]) so that no direct clinical implication can be derived. On the opposite, the reported images have to be regarded as hypothesis-generating for future techniques and devices refinements or educational purposes.

When comparing 3D OCT reconstructions with micro-CT and endoscopy, the limitation of incomplete cross-section visualization due to guidewire shadowing and the possibility of heart-beat related artifacts with OCT might have concurred to some flawed reconstructions.

## Future directions

Multimodal procedural imaging using both standard interventional setting, cutting-edge imaging modality available during clinical practice and high resolution device/anatomy assessments (as done during the present study in the Visible Heart Laboratories) has the potential to impact medical education and device evolution. A series of challenging subsets of complex procedures (ranging between complex stenting techniques like done in the present paper and transcatheter aortic valve implantations as recently reported for the first time [22]) might be assessed by these methodologies. Generated models would in the future be processed by additional computational tool in order to have further insights about mechanical stress, fluid-dynamic reconstructions, interactive didactic material.

## Conclusions

The present study shows the feasibility of real-time multimodal imaging within reanimated swine hearts using Visible Heart® methodologies to assess the specific issues associated with various bifurcation stenting techniques. Detailed device-device and device-tissue interactions were uniquely assessed via tools available in practice today like OCT and compared to live video footages and post-processed micro-CT images collected from perfusion-fixed hearts. These direct comparisons provided unique insights regarding both stent deformations occurring in the course of bifurcation stenting and the efficacy of OCT to visualize them.

## Supplementary Information

Below is the link to the electronic supplementary material.

Supplementary file1 (TIF 1440 KB)

Supplementary file2 (TIF 1494 KB)

Supplementary file3 (DOCX 15 KB)

## Data Availability

All data are included in this report.
