# Supplementary figures and images for: Assessment of single and double coronary bifurcation stenting techniques using multimodal imaging and 3D modeling in reanimated swine hearts using Visible Heart® methodologies
Source: Int J Cardiovasc Imaging. 2021 May 16;37(9):2591–601. doi: 10.1007/s10554-021-02240-0 (PMC8390408; doi:10.1007/s10554-021-02240-0)

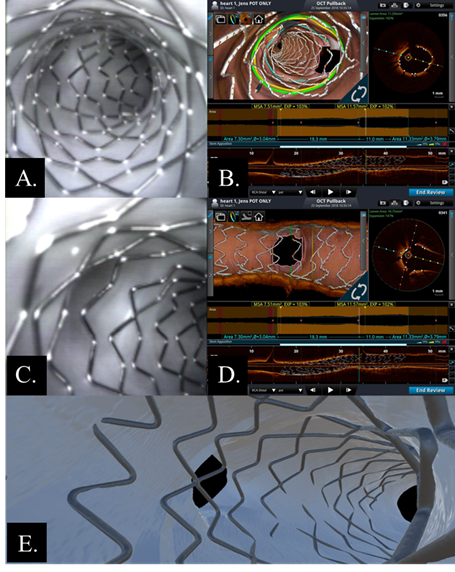

Supplement: Supplementary file 1 — Supplementary file1 (TIF 1440 KB) [file 10554_2021_2240_MOESM1_ESM.tif]

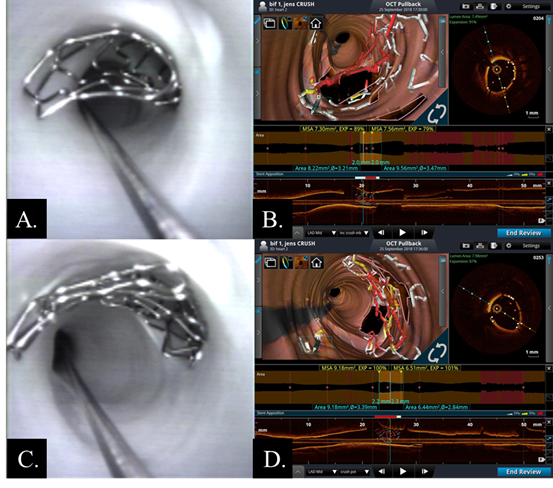

Supplement: Supplementary file 2 — Supplementary file2 (TIF 1494 KB) [file 10554_2021_2240_MOESM2_ESM.tif]
