# Supplementary material for: Assessment of single and double coronary bifurcation stenting techniques using multimodal imaging and 3D modeling in reanimated swine hearts using Visible Heart® methodologies
Source: Int J Cardiovasc Imaging. 2021 May 16;37(9):2591–601. doi: 10.1007/s10554-021-02240-0 (PMC8390408; doi:10.1007/s10554-021-02240-0)
Supplement: Supplementary file 3 — Supplementary file3 (DOCX 15 KB) [file 10554_2021_2240_MOESM3_ESM.docx]

# Supplementary Figure Captions

**Supplementary Figure 1.** Multimodal assessment of results achieved after proximal optimization technique (POT) in provisional. A,B) View of well apposed stent proximal to the bifurcation by direct angioscope and optical coherence tomography (OCT) reconstruction. C,D) Close-up image of bifurcation with angioscope and OCT representation. E) Micro-CT model of stent post-POT, viewed to resemble the 3D OCT reconstruction

**Supplementary Figure 2.** Evaluation of stent crush during Double Kissing (DK)-crush technique. An inefficient crush captured by angioscopic (A) and 3D optical coherence tomography (OCT) reconstruction (B) due to compliancy of the vessels. Angioscopic (C) and 3D OCT reconstruction (D) immediately after performing a secondary crush with a larger sized balloon
